# Lipid droplets and autophagosomes together with chaperones fine‐tune expression of SGK1

**DOI:** 10.1111/jcmm.17300

**Published:** 2022-04-08

**Authors:** Madiha J. Ghani, Wenxue Gu, Zhuyuan Chen, Cecilia M. Canessa

**Affiliations:** ^1^ 12228 School of Medicine Tsinghua University Beijing China; ^2^ Yale School of Medicine Yale University New Haven Connecticut USA

**Keywords:** autophagosomes, CDC37, crosslinking, ER phagy, Hsp90, lipid droplets, SGK1 degradation

## Abstract

Serum‐glucocorticoid‐induced kinase‐1 (SGK1) regulates ion homeostasis and promotes survival under stress conditions. The expression of SGK1 is under transcriptional and post‐translational regulations that are frequently altered in cancer and immune disorders. We report that an N‐terminal amphipathic alpha‐helix determines SGK1 expression levels through two distinct mechanisms. It tethers SGK1 to intracellular organelles generating a large pool of membrane‐bound SGK1, which is differentially stabilized in lipid droplets (LD) in fed conditions or degraded in the endoplasmic reticulum by ER‐phagy in starvation. Association of the α‐helix to organelles does not depend on dedicated receptors or special phospholipids rather, it is intrinsic to its physicochemical properties and depends on the presence of bulky hydrophobic residues for attachment to LDs. The second mechanism is recruitment of protein‐chaperones that recognize the α‐helix as an unfolded protein promoting survival of the cytosolic SGK1 fraction. Together, the findings unveil an unexpected link between levels of energy storage and abundance of SGK1 and how changes in calorie intake could be used to modulate SGK1 expression, whereas the inhibition of molecular chaperones could serve as an additional enhancer in the treatment of malignancies and autoimmune disorders with high levels of SGK1 expression.

## 1 INTRODUCTION

SGK1 is a S/T protein kinase expressed in most mammalian tissues. It protects against numerous stress stimuli^1^, ^2^ and regulates ion homeostasis.^3^ SGK1 weakens self‐tolerance inducing proinflammatory IL‐17 by Th cells^4^, ^5^ and IFNγ in a sodium‐dependent manner.^6^, ^7^ It also contributes to growth and proliferation of cancer cells; specifically, overexpression may take over some Akt1 (PKB) functions to sustain abnormal growth in breast,^8^, ^9^, ^10^ lung^11^ and prostate cancers^2^; and is also essential in Src‐induced transformation.^13^ SGK1 most closely resembles AKT1, both share the same upstream activation signalling pathway and equivalent phosphorylated activation residues but differ in ways intracellular levels are regulated. Unique to SGK1 is a tight control of expression at transcriptional and posttranscriptional levels. Numerous external stimuli (steroids, FSH, p53, TGFβ, UV irradiation and hyperosmotic stress) induce SGK1 transcription.^14^, ^15^ The ubiquitin/proteasomal system degrades SGK1 keeping low steady‐state levels under basal conditions. The short survival depends on amino terminal (Nt) lysines that become ubiquitinated leading to degradation by proteasomes.^16^ Deletion of the Nt extends the half‐life from 28 min to more than 3 h.^17^ However, it is not known whether or how SGK1 protein stability is subject to regulation under physiological and pathological conditions.

Driven by the frequently increased expression level of SGK1 in pathologies, particularly in cancer and T cell‐mediated disorders, we sought to identify post‐translational processes and interacting proteins that modulate the abundance of SGK1 in cells and in mouse tissues. We found two distinct processes operating alongside that either stabilize or promote degradation of SGK1. One relies on SGK1 association to intracellular organelle's membranes and the other recruits a large array of chaperones from different protein quality control machineries. These regulatory mechanisms could be targeted for therapeutic reduction of SGK1 levels in malignant cells and other pathological processes that exhibit increased SGK1 transcription.

## 2 MATERIALS AND METHODS

Supplementary File 1.

## 3 RESULTS

### 3.1 Hydrophobicity of the Nt of SGK1 drives association to liposomes in vitro and to membranes of organelles in cells

**FIGURE 1:**
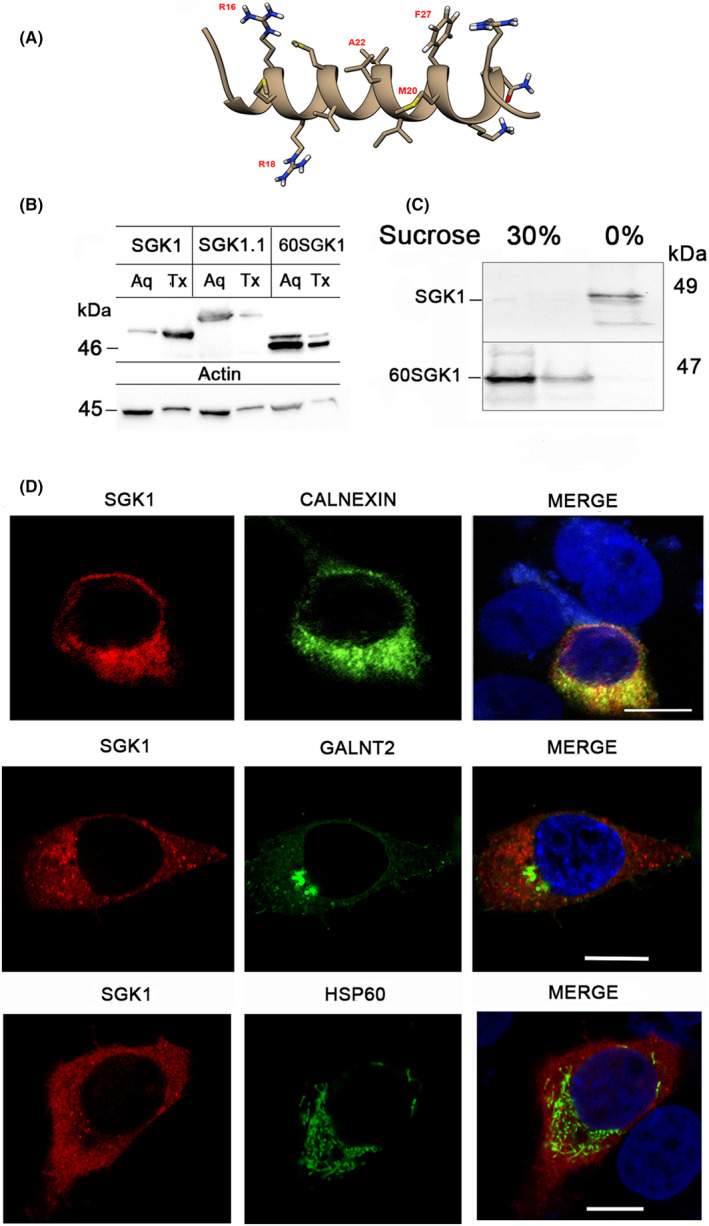
Amino acid sequence of hSGK1 aminoterminus predicts a hydrophobic α‐helix that binds to lipid membranes. (A) Secondary structure of hSGK1 from residue R16 to R31 predicted by PEP‐FOLD. Ribbon representation of backbone with side chains shown as sticks using UCSF Chimera software. (B) Phase separation of cell lysates with TritonX‐114 shows SGK1 preferential partitions in the detergent phase whereas ∆60SGK1, SGK1.1 and actin partition in the aqueous phase (Aq). Equal amount of protein was loaded in each lane. (C) Reconstituted recombinant SGK1 and ∆60SGK1 into artificial liposomes loaded on top of a 0 to 30% sucrose gradient. SGK1 was recovered after gradient centrifugation from the top layer (0% sucrose) that contains proteins integrated in liposomes. ∆60SGK1 was found at the bottom of the gradient (30% sucrose) that contains proteins not incorporated in liposomes. (D) Confocal immunofluorescence images of HEK293T expressing SGK1‐FL with exogenous calnexin‐V5, GALNT2‐HA and Hsp60‐HA used to mark ER, Golgi apparatus, and mitochondria, respectively. Quantification of colocalization shown in Table S3. ×100; scale bar 10 µm. Each experiment was repeated 3 times

The crystal structure of human SGK1 catalytic domain has been solved (PDB ID: 3HDM) but not that of the Nt that encodes a critical degron. Prediction of the secondary structure of a stretch of hydrophobic residues using PEP‐FOLD yields an α‐helix from residues R16 to R31 (Figure 1A). As the length of the helix is too short to span cellular lipid bilayers, it remains exposed as a hydrophobic patch after the synthesis and folding of the kinase domain have been completed; thus, the Nt may challenge survival of the protein acting as a degron. To confirm experimentally the hydrophobicity predicted in silico, we first tested Triton X‐114 phase partitioning of SGK1.^19^ SGK1 primarily is in the Triton X‐114 phase but after the deletion of the Nt, the truncated protein (∆60SGK1) partitions to the aqueous phase (Figure 1B). SGK1.1—a spliced isoform differing only in the amino terminal sequence‐^22^ also partitions to the aqueous phase indicating that the predicted α‐helix exhibits significant intrinsic hydrophobicity.

Next, we examined whether SGK1 associates to artificial lipid bilayers by incorporating recombinant SGK1 and ∆60SGK1 into artificial liposomes examined by floatation through a sucrose step gradient. Figure 1C shows a Western blot of SGK1 recovered from a 30 to 0% sucrose gradient. SGK1 was found in the top liposome‐containing fraction whereas ∆60SGK1 was in the bottom fraction, indicating that the Nt is required for SGK1 association to lipid bilayers and the association does not need specialized phospholipids or intrinsic membrane protein receptors. Thus, physicochemical properties of the Nt are sufficient and necessary for association to artificial lipid membranes.

We next looked for association of SGK1 to cellular membranes using immunofluorescence microscopy. The endoplasmic reticulum (ER) was identified with calnexin, the Golgi apparatus with polypeptide N‐acetylgalactosaminyltransferase 2 (GALNT2) and mitochondria with Hsp60. SGK1 colocalized extensively with ER, some with the Golgi complex and was undetected in mitochondria (Figure 1D), showing that SGK1 has also affinity to cellular membranes.

### 3.2 Binding to lipid droplets stabilizes and extends the half‐life of SGK1

**FIGURE 2:**
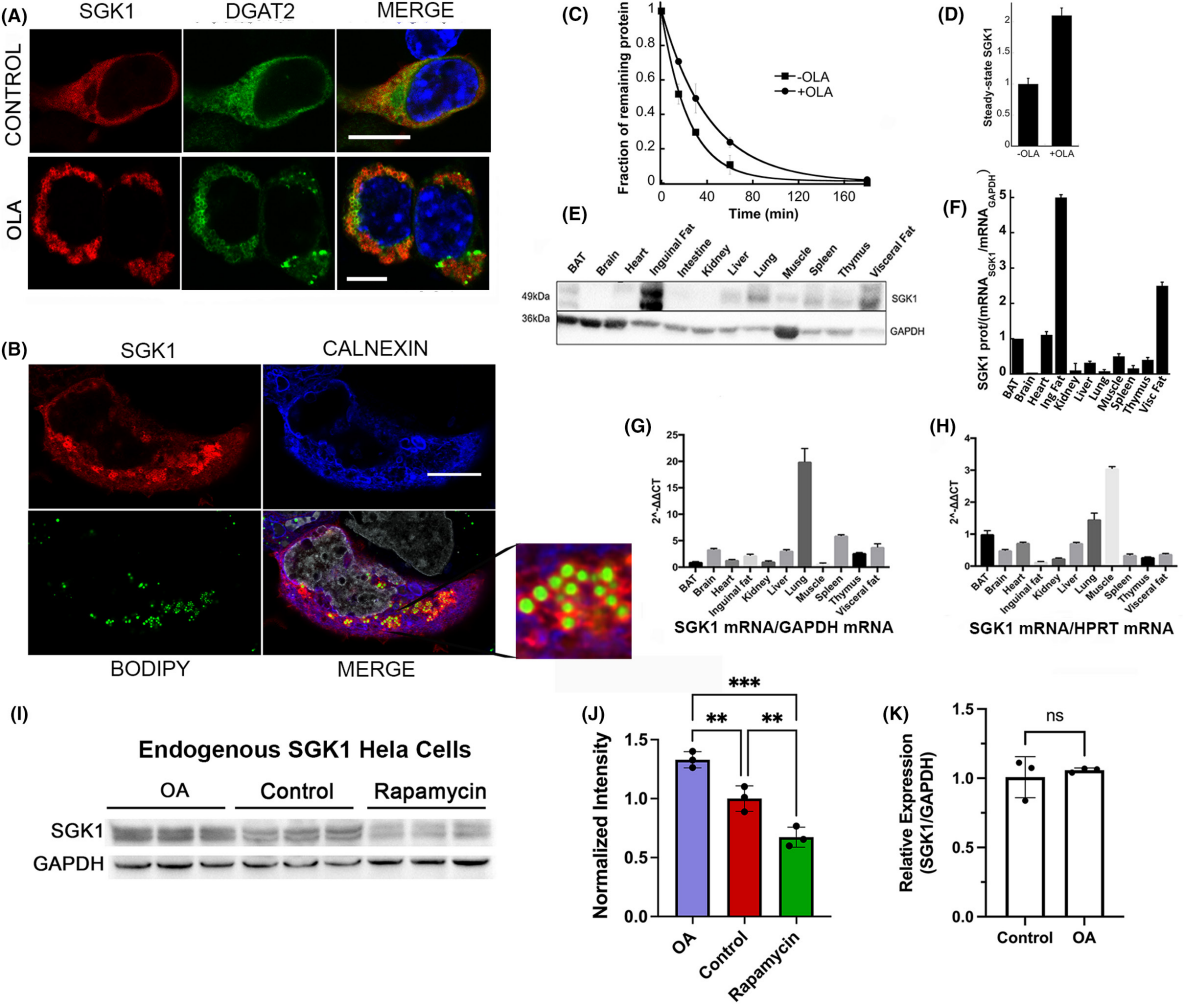
Association to lipid droplets stabilizes SGK1. (A) Confocal images of cells expressing SGK1 or DGAT2 in standard medium (control) and supplemented with oleic acid (OLA). LDs are visualized with BODIPY 480/515 (green). Merge images include staining of DNA with DAPI (blue). (B) Confocal images of cells expressing SGK1 treated with OLA. SGK1 is shown in red, the ER marker Calnexin is in blue, LD are stained with BODIPY in green, and nuclei stained with DAPI are shown in grey. Magnification of a cytosolic area containing LDs shows SGK1 surrounding LD but not Calnexin. Magnification: x100. Scale bars: 10 µm. Quantification analysis of colocalization of A and B are shown in Table S3. (C) Plots of SGK1 half‐life ±OLA. Each data point is the mean of 3 independent pulse‐chase experiments with [S^35^]methionine/[S^35^]cysteine. Original gels shown in Figure S1A. (D) Quantification of steady‐state abundance of SGK1 ± OLA estimated from band intensity of SGK1 over actin in western blots (*n* = 3). (E) Representative western blot of SGK1x3FL abundance in various mouse tissues from a transgenic line that expresses SGK1‐FL in all tissues under the CAG promoter. Equal amount of protein was loaded in each lane. GAPDH antibody was used as internal control. Design and generation of transgenic mouse shown in Figure S2. (F) Ratio of SGK1 protein per ratio of SGK1_mRNA_/GAPDH_mRNA_ in the indicated tissues. (G) and (H) qPCR analysis of SGK1 transcript abundance in same mouse tissues normalized to GAPDH or HRPT transcrips. Experiments were repeated three times. (I) Steady‐state abundance of endogenous SGK1 in Hela cells control and treated with OA or Rapamycin for 6 h. (J) Graph is the analysis of the three corresponding samples shown in the western blot normalized to control. Statistical differences evaluated with one‐way‐ANOVA and Tukey's multiple comparison test; *p* ** 0.009, *** 0.0002. (K) qPCR of Hela ±OA. *T*‐test: no significant difference (ns)

We have previously shown that SGK1 is ubiquitinated by the Ub‐conjugating UBC6 and UBC7 enzymes and the Ub ligase Hrd1 that are ER‐associated and lead to rapid SGK1 degradation.^16^, ^17^ The ER gives rise to LDs that are surrounded by a single layer of phospholipids.^23^ Many proteins targeted to LDs often contain amphipathic helices^24^ raising the possibility that the Nt could target SGK1 to LDs. The hydrophobic fluorescent dye BODIPY (493/503) or co‐transfection with DGAT2 (diacylglycerol O‐acyltransferase)^25^ was used to identify LDs. In 293T fed with standard medium, DGAT2 was anchored to the ER membrane but after incubation with oleic acid (OLA), numerous LDs were formed and DGAT2 changed localization from the ER to the LDs. After OLA treatment, SGK1 also decorated LDs though, a large fraction remained associated to the ER colocalized with calnexin (Figure 2A–B). Notably, the presence of LDs increased total SGK1 abundance by a factor of 2 and lengthened the half‐life from 24 to 42 min measured by pulse‐chased and cycloheximide chase experiments (Figure 2C–D, Figure S1).

The LD‐mediated stabilization of SGK1 observed in cultured cells was confirmed in tissues of transgenic mice that express SGK‐3FL driven by the CAG promoter (Figure S2). We used these mice because the low expression of endogenous SGK1 in tissues makes it difficult to detect the protein as demonstrated by Western blots of heart and skeletal muscle (Figure S3A). In addition, the abundance of SGK1‐3FL transcript in transgenics is not influenced by the numerous factors that induce transcription of the endogenous gene; thus, only posttranscriptional rate of degradation but not transcription impacts SGK‐3FL abundance. Western blots show that white‐fat tissues exhibit the highest expression level of SGK1‐3FL (Figure 2E–F). To quantify mRNA levels of SGK1‐3FL, the same tissues were examined by qPCR. Given the variable expression of GAPDH among tissues, data were normalized with two different housekeeping genes: GAPDH and HPRT (Figure 2G–H). In both cases, the ratio of SGK1‐3FL protein to mRNA was highest in adipocytes consistent with SGK1 stabilization in LD containing tissues.

To probe whether LDs also stabilize the endogenous SGK1 protein, Western blot analysis of HEK293T was conducted with a rabbit monoclonal anti‐SGK1 antibody; however, the signal was very weak even after the induction of SGK1 transcription with 100 µM dexamethasone for 2 h.^14^ We next screened tumour derived cells as high SGK1 expression has been reported in numerous cancer types.^8^, ^9^, ^10^, ^11^, ^12^ Western blot analysis of several cell lines identified Hela as the one with highest level of SGK; hence, Hela cells were chosen to examine the stability of endogenous SGK1 by cycloheximide chase: the calculated t_1/2_ was 42 min (Figure S2B–C); and induction of LD increased the steady‐state abundance of endogenous SGK1 without changing transcription measured by qPCR (Figure 2I–K). Together, these results are consistent with post‐translational stabilization as the main mechanism whereby LD increase SGK1 abundance of the endogenous and transfected SGK1 in cultured cells and in mouse tissues.

### 3.3 **Large hydrophobic side chains in the** α**‐helix of SGK1 are necessary for association with LDs but not to other intracellular membranes**

**FIGURE 3:**
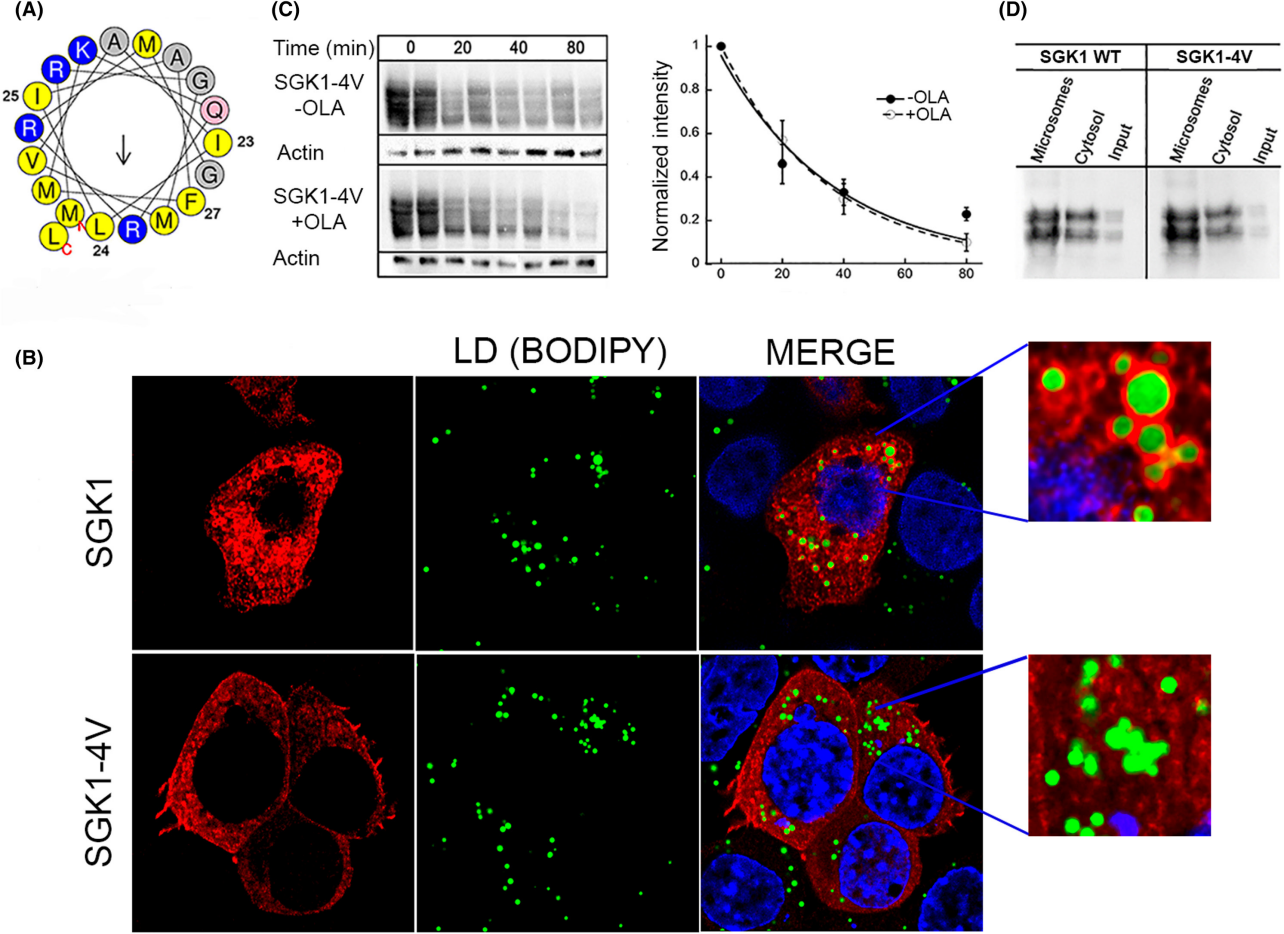
Large hydrophobic side chains in SGK1 α‐helix are necessary for association to lipid droplets. (A) Helical wheel representation of the α‐helix using HeliQuest program. Mean hydrophobicity 0.595, hydrophobic moment 0.31. After residues I23, L24, I25, and F27 were mutated to valine the values of mean hydrophobicity and hydrophobic moment changed to 0.48 and 0.27. (B) Confocal images of 293T expressing SGK1 (upper panels) or SGK1‐4V (lower panels) grown in medium supplemented with OLA stained with BODIPY (green) and anti‐FL monoclonal to detect SGK1 and SGK1‐4V. Only SGK1 forms a ring around LDs whereas SGK1‐4V does not colocalize with LDs. Nuclei stained with DAPI shown in blue. Magnification: x100, scale bars: 10µm. (C) Western blot of cycloheximide‐chase experiments conducted with SGK1‐4V ± OLA in the medium. Graph represents the time course of SGK‐4V decay analyzed by immunoblotting. Lines are the fit of data to a single exponential with *t_1_*_/_ *_2_* of 34 and 37 min. (D) Western blot of the distribution of SGK1 and SGK‐4V in microsomal and cytosolic fractions of cells grown in standard medium after homogenization and high‐speed centrifugation. Equal amount of protein was loaded from each fraction. Input represents 1/10 of the sample prior to fractionation by centrifugation. Each transfection was performed in duplicate and experiments repeated three times

Previous studies have shown that proteins with large hydrophobic residues in amphipathic helices preferentially bind to the surface of LDs.^24^ To test if this principle applies to SGK1, we substituted four bulky hydrophobic side chains: I23, L24, I25 and F27 for valine to generate the construct SGK1‐4V. Figure 3A shows a helical wheel representation of SGK1 α‐helix and positions of the four substituted residues. SGK1‐4V expressed in 293T grown in medium supplemented with OLA did not bind to LDs in contrast to wildtype SGK1 that encircled LDs consistent with the requirement of bulky hydrophobic side chains in the α‐helix to bind LDs. The half‐life of SGK1‐4V examined by pulse‐chase in cells treated with or without OLA was found to be *t_1_*_/_ *_2_* 34 and 37 min similar to that of wild type SGK1, and the distribution of SGK1‐4V between microsomes and cytosol in standard medium was not altered, indicating that binding to other microsomal membranes was not compromised (Figure 3B–D). Thus, bulky hydrophobic side chains play a crucial role in tethering SGK1 to LD but they are not essential for binding to other organelles.

### 3.4 Starvation transfers a fraction of SGK1 to phagosome/lysosome degradation

**FIGURE 4:**
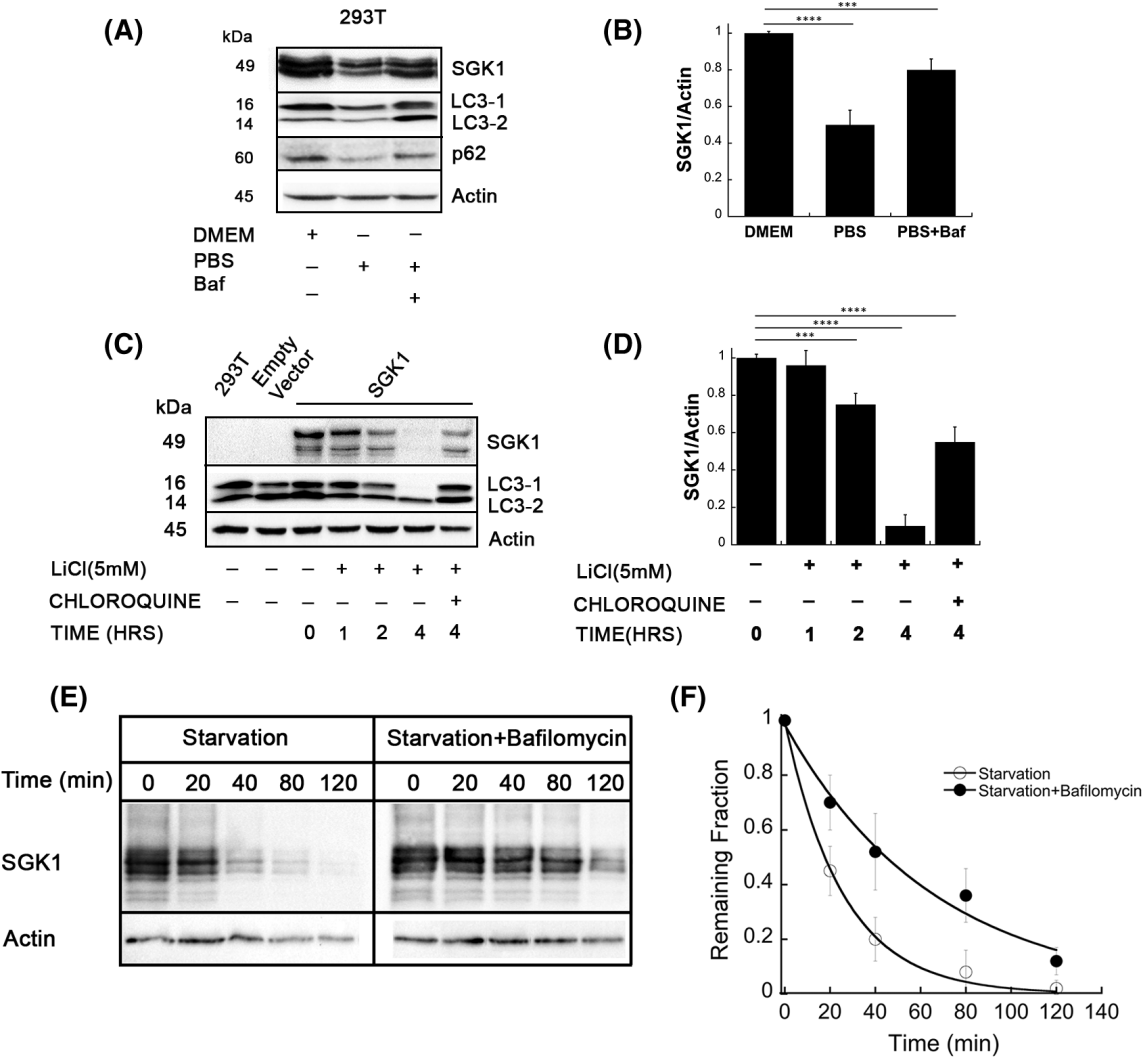
Starvation‐induced autophagy decreases abundance of SGK1. (A) Western blots of SGK1 and LC3 in 293T control and treated with PBS for 4 h. Markers of autophagy: conversion of LC3‐1 to LC3‐2, accumulation of LC3‐2 by bafilomycin and chloroquine, and reduction of p62. (B) Bars represent quantification of SGK1 normalized to actin, *n* = 3, *p‐value *< 0.01. (C) Western blots of SGK1 and LC3 of cells treated with LiCl to induce autophagy and chloroquine to block lysosomes. (D) Bars represent quantification of SGK1 normalized to actin in the indicated conditions. *n* = 3; *p*‐*value *< 0.01. (E) Representative pulse‐chase of 293T expressing SGK1 treated with PBS for 4 h ±bafilomycin and chased for 2 h. (F) Each data point in the graph represents the mean of three independent blots. The curve is the fit of the data to a single exponential with derived *t_1_*_/_ *_2_* of 20 ± 3. min and 40 ± 10 min

Treatment with phosphate saline for 4 h ±bafilomycin or chloroquine (inhibitors of lysosomes) decreased SGK1 expression by 50 ± 6% (Figure 4A–B). Starvation is a physiological trigger of autophagy and since many autophagosomes originate from ER membranes, we looked for the possibility of SGK1 association to phagosomes as a cause of decreased SGK1. Because starvation inhibits the mammalian target of rapamycin complex 1 (mTORC1) inducing autophagy but also represses protein synthesis, we sought to differentiate the contribution of these two processes on SGK1 expression. The distinction is relevant because steady‐state abundance of short‐lived proteins such as SGK1 is highly sensitive to suppression of synthesis. We used LiCl, which induces autophagy independent of mTORC1.^26^ Conversion of LC3‐1 to LC3‐2 shows that LiCl induces autophagy and decreases SGK1 (Figure 4C‐D). Starvation shortens the half‐life (*t_1_*_/_ *_2_* 20 ± 5 min) and lysosomal inhibitors (*t_1_*_/_ *_2_* 40 ± 8 min) stabilizes it (Figure 4E–F), suggesting that stimulation of autophagy by means of starvation or LiCl‐ redistributes a fraction of SGK1 to autophagosomes/ lysosomes decreasing SGK1 abundance.

**FIGURE 5:**
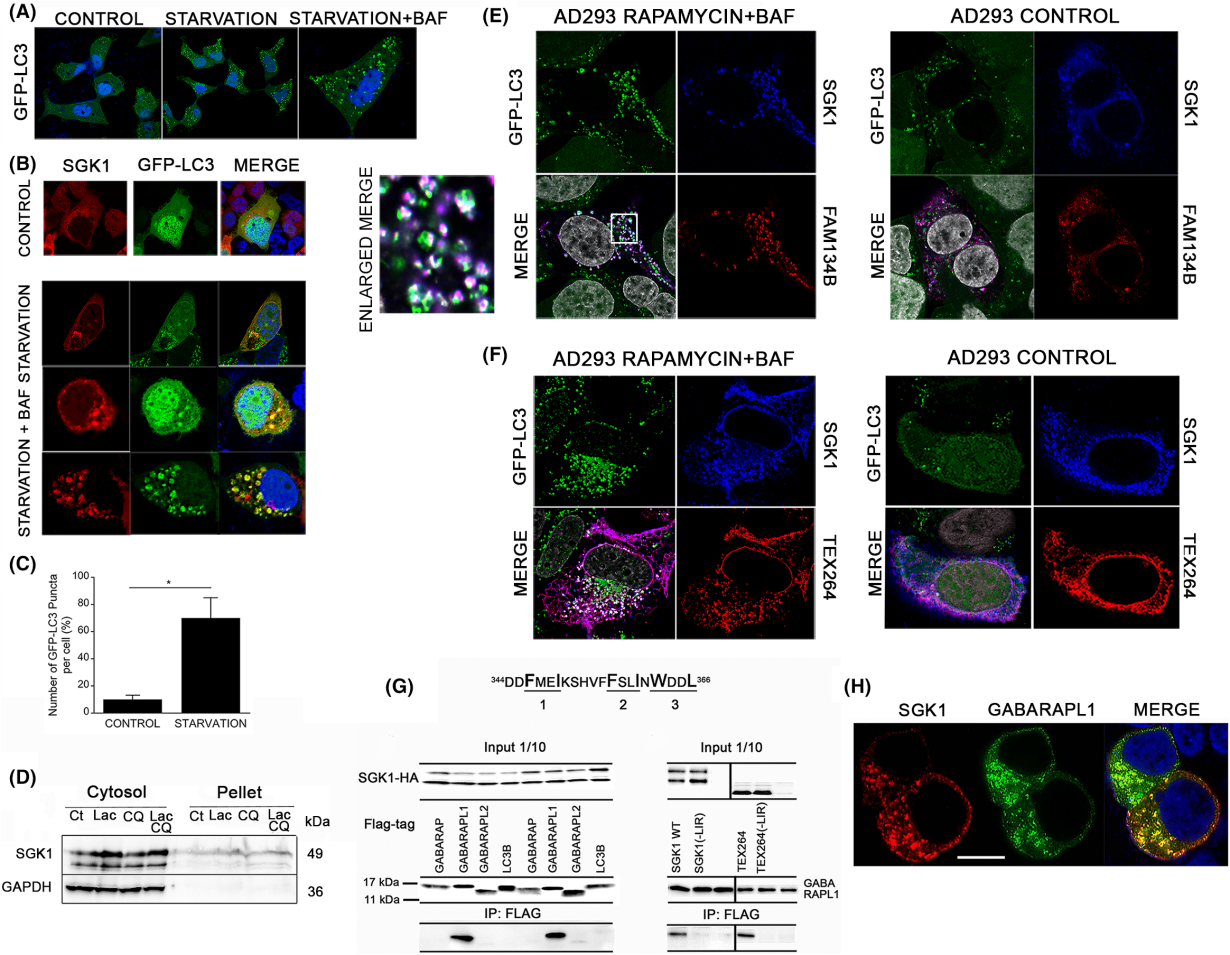
SGK1 enters autophagosomes in starved cells together with LC3 and specific ER‐phagy receptors. (A) AD293 cells stably transfected with GFP‐LC3 in control medium, after starvation (rapamycin 0.5 µM for 4 h), and starvation in the presence of 0.1µM bafilomycin A (BAF) (third panel enlarged five‐fold). (B) Confocal images of AD293‐GFP‐LC3 cotransfected with SGK1‐HA (red) treated with control medium, starvation (rapamycin 0.5 µM for 4 h), and starvation +BAF (two lower rows). (C) Quantification of the number of GFP‐LC3 puncta/vesicles per cell under control and after starvation conditions analysed using the “Analyze Particles” function. Asterisks = *t*‐test, *p* value < 0.001. (D) Western blot of SGK1 distribution in soluble (supernatant) and insoluble (pellet) fractions of cell lysates solubilized with 1% SDS after treated with 5 µM lactacystin (5 µM Lac), chloroquine (10 µM CQ) and both. (E) Confocal images of AD293‐GFP‐LC3 co‐transfected with SGK1‐HA (blue) and FAM134B‐FL (red) treated with Bafilomycin in the presence of 0.1 µM BAF (starvation condition) compared to control. White puncta indicate colocalization of the three indicated proteins. Nuclei stained with DAPI are shown in grey. Enlarged merge: ten‐fold enlargement of the area delimited by the square. (F) AD293‐GFP‐LC3, expressing SGK1‐HA (blue) and TEX264‐FL (red) under basal condition or rapamycin‐induced starvation plus BAF to induce autophagy. White puncta in merge image represent colocalization of the three indicated proteins. Nuclei are shown in grey. (G) SGK1 sequence from amino acid 344 to 366 encodes three putative LIR motifs (underlined). Co‐immunoprecipitation of SGK1‐HA and mammalian Atg8 proteins (LC3B, GABARAP, GABARAPL1 and GABARAPL2) tagged with FL. 1/10 of the input was analysed by Western blotting. IP conducted with anti‐HA magnetic beads and developed with anti‐FL antibody. The blot shows two independent experiments. Co‐immunoprecipitation of SGK1‐HA wildtype, SGK1‐HA with three mutations that eliminate the putative LIR consensus sequences (‐LIR), TEX264‐HA and TEX264(‐LIR)‐HA together with GABARAPL1. Blot shows input and co‐IP results. (H) Confocal image of SGK1‐HA and FL‐GABARAPL1 in 293T. x100; scale bar 10 µm. Quantification analysis of colocalization of E, F and H images are shown in Table S3

To examine whether SGK1 localizes in autophagosomes, we used AD293 cells stably transfected with LC3 fused with GFP (GFP‐LC3). Starvation induced by removing serum and adding rapamycin for 4 h led to the redistribution of the green fluorescence signal from a diffuse pattern into puncta that grew to the size of large vesicles after treatment with the lysosomal inhibitor bafilomycin A1 (BAF), consistent with the vesicles being autophagosomes (Figure 5A). These cells were transfected with SGK1 and examined under control and rapamycin‐induced in the presence of bafilomycin. Control panel of Figure 5B shows diffuse GFP‐LC3 in cytosol and nucleus whereas after starvation, colocalization with SGK1 was detected in many small puncta that enlarged after treatment with bafilomycin. Quantifications of the overlap of SGK1 and GFP‐LC3 and total number of puncta are shown in Figure 5C.

To answer how SGK1 could end up in autophagosomes, we first examined whether SGK1 forms cytosolic insoluble aggregates captured by autophagosomes.^27^, ^28^ Cells were treated with the proteasome inhibitor lactacystin with or without chloroquine for 6 h; after lysis with 1% Triton‐X100, the presence of SGK1 was examined in the supernatant and pellet by immunoblotting. The intensity of SGK1 signal increased with inhibitors but the protein was mostly recovered from the soluble cytosolic fraction, indicating that even after ubiquitination SGK1 does not form a significant number of insoluble aggregates thus, autophagy of cytosolic SGK1 does not seem to be the main degradation pathway (Figure 5D). As SGK1 localizes to ER membranes from where it could be transferred to emerging phagophores or engulfed together with ER membrane fragments by ER‐phagy, we examined the co‐expression of SGK1 with two ER‐resident autophagy receptors FAM134B^29^ or TEX264.^30^, ^31^ We observed broad colocalization of SGK1 with FAM134B or TEX264 in the ER and nuclear envelop (Figure 5E–F). In basal conditions, a small degree of ER fragmentation was observed as previously reported,^29^, ^30^ whereas cells treated with rapamycin together with bafilomycin displayed extensive ER fragmentation and large vesicles containing GFP‐LC3, SGK1 and FAM134 (Figure 5E) or TEX264 (Figure 5F), indicating that SGK1 and ER fragments are engulfed together by phagosomes (shown as white puncta/vesicles in the merged panels of rapamycin+baf treated cells (Figure 5E–F).

These findings suggest that starvation‐induced ER‐phagy sends a fraction of SGK1 to autophagosomes for lysosomal degradation. The mechanism by which SGK1 ends in autophagosomes could be proximity to ER‐phagy receptors that is, SGK1 hitchhikes its way to autophagosomes or alternatively, autophagic membranes could also recognize directly SGK1 in the ER surface. We noticed three putative motifs (the first one conserved from frog to human) (Figure 5G) in the carboxyterminus of SGK1 that conform to the sequence LC3‐interacting regions (LIRs) or GABARAP‐interacting motifs (GIMs) shared among autophagy adaptors and receptors. The canonical LIRs and GIMs consist of a consensus motif [W/F/Y]xx[L/I/V] surrounded by at least one proximal acidic residue.^32^ To investigate if such motifs play a role in recruiting SGK1 into autophagosomes, we conducted co‐IPs of SGK1‐HA and FL‐GABARAPs. FL‐GABARAPL1 and small amount of FL‐GABARAPL2 but no LC3B was pulled down (Figure 5G). When the putative LIR motifs of SGK1 were mutated—F346A/F354A/W360A—the co‐IP failed to pull down FL‐GABARAPL1, suggesting that SGK1 carboxy‐terminus may facilitate recruitment by interacting with specific adaptors present in phagosomes. SGK1 and GABARAPL1 also colocalized extensively by immunofluorescence (Figure 5H) whereas GABARAP and GABARAPL2 showed homogeneous cytosolic distribution without colocalization with SGK1 in membrane compartments (data not shown).

### 3.5 **SGK1 recruits chaperones from many classes of protein quality control systems**.

Thus far, we have shown that SGK1 associates to various intracellular organelles and SGK1 stability is organelle‐specific. However, the cytosolic fraction of SGK1 is also highly susceptible to degradation. To identify proteins that promote or protect SGK1 from degradation, we used crosslinking strategies in 293T expressing SGK1. First, we used disuccinimidyl suberate (DDS) (Figure S3A). Table 1 lists identified proteins with respective abundance scores. The highest hits were protein‐folding chaperones from the heat shock family: Hsp70 and Hsp71 with their cochaperones (DnaJ/Hsp40); Hsp90α/β and their cochaperones CDC37; subunits of the eukaryotic chaperonin Hsp60; chaperonin containing TCP‐1; ATP‐independent molecular holdases: Bat3/Bag6 (BCL2 associated athanogene 6) and SGTA (small glutamine‐rich tetratricopeptide repeat‐containing protein α). To examine whether chaperone recruitment requires the Nt of SGK1, similar crosslinking was conducted with the N‐terminal deletion (∆60SGK1). Fewer chaperones were recovered despite ∆60SGK1 expressing ~5‐fold higher level than wildtype (Table S1). To further confirm and expand these results, we used a photo‐crosslinker: photo‐leucine^33^ (Table S2). The number of crosslinked proteins was four times lower than that obtained with DSS, but the top hits were almost identical.

**TABLE 1:** SGK1 binding proteins identified by mass spectroscopy

SGK1			
DSS		CONTROL	
Protein	Score	Protein	Score
Hsp 70 1A	2344.63	Hsp 70 1A	154.77
**SGK1**	2161.81	**SGK1**	1178.33
Hsp cognate 71	2102.15	Hsp cognate 71	57.60
Hsp 60	1838.06		
Hsp 90‐b	1227.47	Hsp 90‐b	35.06
CDC37	1153.20	CDC37	36.08
Hsp 90‐a	1142.54		
DnaJ homolog subfamily A1	297.07		
FKBP4	283.83		
T‐complex protein 1 subunit a	151.32		
BAT3	150.50		
Hsp 75 mitochondrial	120.09		
T‐complex protein 1 subunit b	105.14		
SGTA	104.91		
Hsp70‐interacting protein	78.61		
T‐complex protein 1 subunit q	68.38		
T‐complex protein 1 subunit g	58.15		
Peptidyl‐prolyl cis‐trans isomerase	56.28		
T‐complex protein 1 subunit h	55.52		
T‐complex protein 1 subunit z	45.59		
T‐complex protein 1 subunit d	39.50		
Nascent polypeptide‐associated complex subunit a	38.85		
T‐complex protein 1 subunit e	30.92		
FKBP8	20.19		
DnaJ homolog subfamily C7	13	DnaJ homolog subfamily C	1.69
Hsp 70 4	10.20		
FKBP5	2.89		
DnaJ homolog subfamily B1	2.33		
DnaJ homolog subfamily A2	1.71		

Note

First column lists proteins immunoprecipitated with SGK1 after crosslinking with SSD whereas control indicates no crosslinking reagent. The score relates to the abundance of the recovered protein.

**FIGURE 6:**
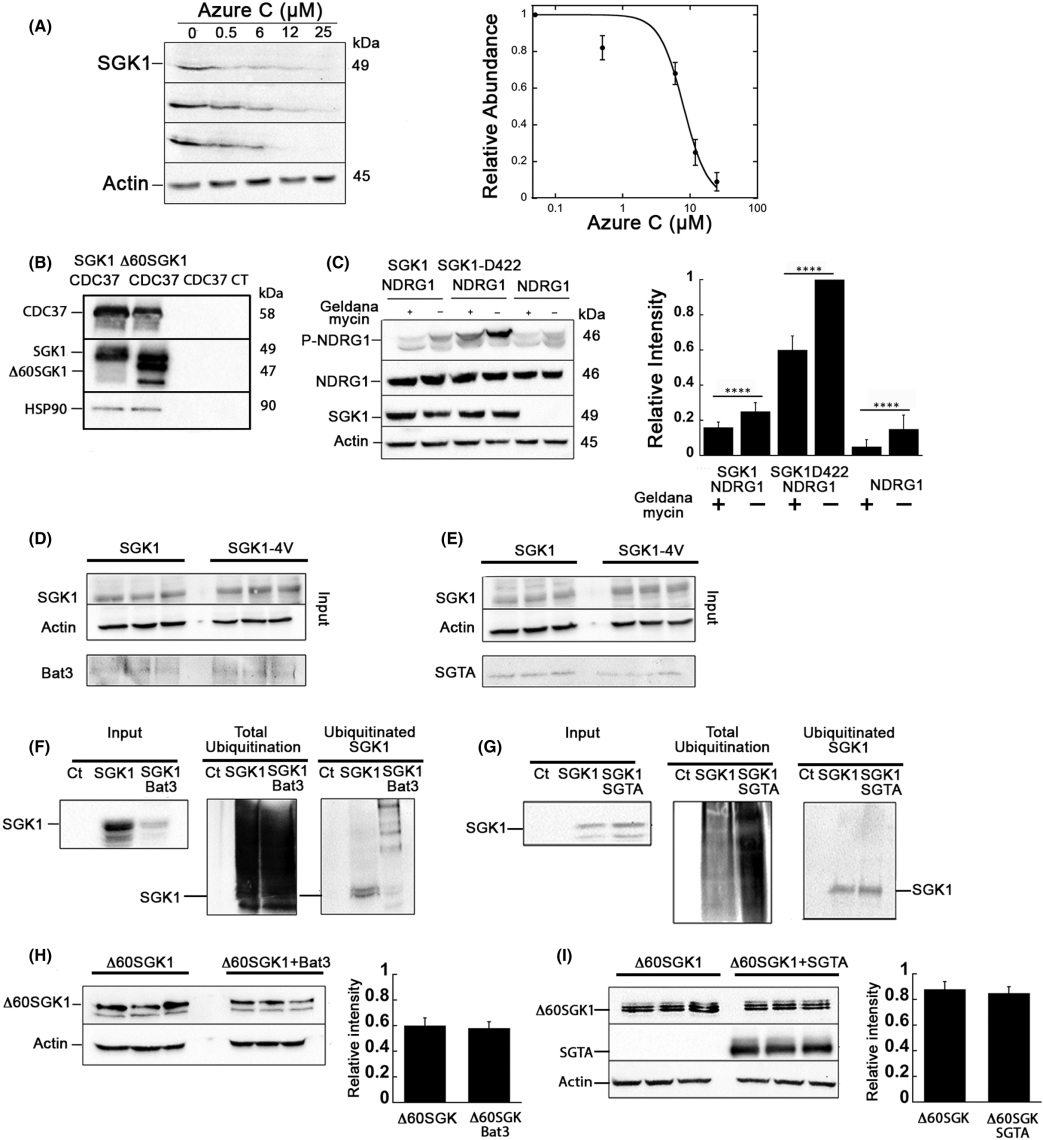
Effects of protein chaperones on SGK1 abundance and function. (A) Representative western blot of three experiments of SGK1 expressed in 293T cells treated with increasing concentrations of Azure C for 3 h. Concentration‐response curve and estimated apparent inhibitory concentration of Azure C, IC_50_ = 8 µM. Each data point is the mean ± SD of the intensity of SGK1 bands shown in the Western blots. (B) Cells expressing CDC37‐Flag with SGK1‐HA, ∆60SGK1‐HA or no SGK1 were co‐precipitated using HA monoclonal. The images show the elution blotted with HA, Flag and endogenous Hsp90, indicating the three proteins form a complex. CT = control non‐transfected cells. (C) Geldanamycin inhibition of Hsp90 decreases SGK1 mediated phosphorylation of NDRG1, a target phosphorylated SGK1. The constitutively active form SGK1‐S422D markedly magnifies the effect. Columns represent the mean ± SD, (*n* = 3); asterisks indicate statistically significant differences between conditions with *p*‐values < 0.01. (D) Representative western blot of three experiments where SGK1 and SGK1‐4V co‐immunoprecipitate endogenous Bat3. (E) Representative western blot of three experiments where SGK1 and SGK1‐4V co‐immunoprecipitate SGTA. (F) Left Panel: Input. Equal amount of protein from control cells (non‐transfected) and cells transfected with SGK1 alone or with SGK1 and Bat3 examined with anti‐SGK1 antibody. Middle Panel: All ubiquitinated proteins. Cell lysates affinity purified with TUBE resin for isolation of all ubiquitinated proteins and blotted with anti‐ubiquitin antibody. Ct = no TUBE resin. Right Panel: Ubiquitinated SGK1. Proteins isolated with TUBE blotted with anti‐SGK1 antibody. (G) Similar experiment as describe in F but here cells were co‐transfected with SGK1 and SGTA. (H) Expression level of ∆60SGK1 in cells co‐transfected ±Bat3 and quantification mean ± SD (*n* = 3). (I) Expression level of ∆60SGK1 in cells co‐transfected ±SGTA and quantification mean ± SD, (*n* = 3). Each transfection was performed in triplicate, experiments were repeated three times

Functional significance of SGK1 interactions with crosslinked chaperones was examined. Suppression of endogenous Hsp70 activity by the inhibitor Azur C destabilized SGK1 significantly reducing steady‐state abundance (Figure 6A). Hsp90/CDC37 was co‐immunoprecipitated with both SGK1 and ∆60SGK1 (Figure 6B) and inhibition of Hsp90 with the inhibitor geldanamycin decreased the catalytic activity of SGK1 demonstrated by decreased NDRG1 phosphorylation, which is a physiological substrate of SGK1 (Figure 6C).^34^ These experiments indicate that Hsp proteins and their co‐chaperons stabilize SGK1 and that recruitment of Hsp90/CDC37 is independent of the Nt consistent with this complex binding to the catalytic domain as previously reported for other kinases of the AGC family.^35^, ^36^

**FIGURE 7:**
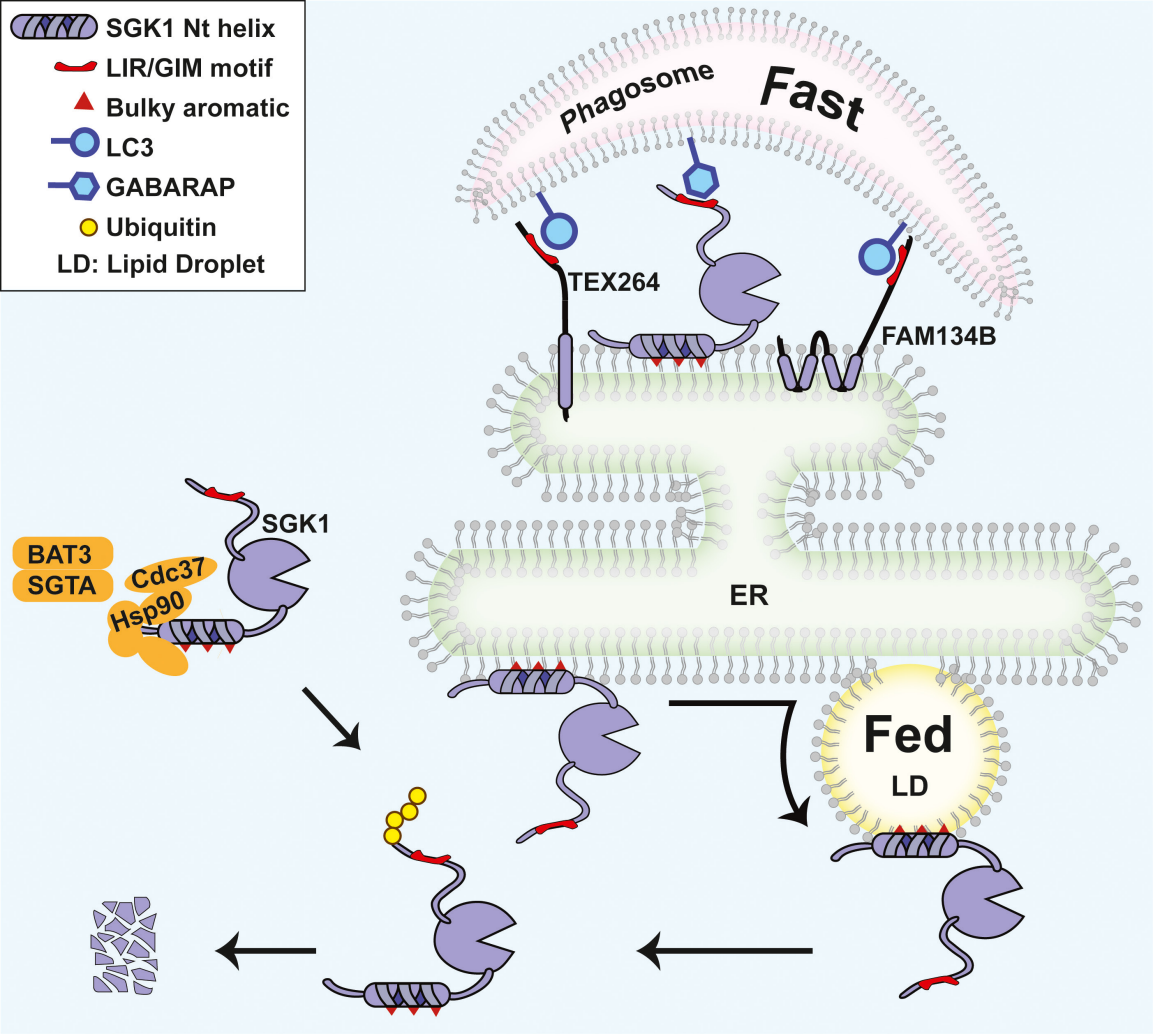
Post‐translational mechanisms modulate SGK1 protein abundance. Hydrophobicity of the Nt α‐helix of SGK1 attracts chaperones from various protein control systems to protect SGK1 from ubiquitination and degradation. The hydrophobic environment of lipid membranes also drives localization of SGK1 to organelles where it is stabilized in lipid droplets (LD), degraded in lysosomes if transferred from the ER to autophagosomes, or subject to the endoplasmic reticulum associated degradation (ERAD) while remaining associated to the cytosolic leaflet of the ER. Fasting and energy surplus impact SGK1 stability in opposite directions, fine‐tuning SGK1 levels in various physiological and pathological states

SGTA and Bat3/Bag6 have been linked to the degradation of misfolded proteins; they form a complex together with UBL4A and TRC35 that specializes in preventing the aggregation of transmembrane segments of integral membrane proteins that are either mislocalized or retrotranslocated from the ER to the cytosol.^37^, ^38^, ^39^ They also assist posttranslational delivery of tail‐anchored membrane proteins for insertion in the ER membrane.^40^ Such clients have in common a hydrophobic α‐helix exposed to the cytosol similar as the Nt of SGK1, although in the instance of SGK1 the hydrophobic α‐helix is not long enough to traverse lipid bilayers. If refolding of the client fails, the Bat3/Bag6 complex binds a ubiquitin ligase (RNF126) that ubiquitinates and transfers the client to the proteasome. We found that SGK1 and SGK1‐4V associate with endogenous Bat3/Bag6 in 293T but not with ∆60SGK1, concordant with the crosslinking experiments. The overexpression of Bat3/Bag6 reduced levels of SGK1 owing to increased ubiquitination whereas ∆60SGK1 levels were not altered (Figures 6, 7). SGTA co‐immunoprecipitated with SGK1 and SGK1‐4V, decreased ubiquitination and increased the total amount of SGK1, and that these effects required presence of SGK1 α‐helix (Figures 6, 7). The results show that even though SGK1 is not a typical Bat3/SGTA client, they bind the amphipathic α‐helix and modulate SGK1 stability.

## 4 DISCUSSION

Tight control of intracellular protein levels is necessary to adjust cellular responses to rapidly changing external and internal stimuli in order to adapt to fluctuations in the environment. This requirement cannot be met only by transcriptional regulation but also requires modulation of the target protein half‐life. Unique among the AGC family of kinases, SGK1 is subject to fine‐tuning of its intracellular concentration by transcription and degradation, a precondition necessary to modulate the response of SGK1 and integrate it with the signals of overlapping kinases such as AKT1.

The main protein degradation pathways in eukaryotes are the proteasome, lysosome and autophagosomes. Conventionally, the ubiquitin‐proteasome degradative system is primarily responsible for the turnover of short‐lived proteins whereas autophagy degrades the bulk of long‐lived cytosolic proteins though, these differences may blur as more proteins are examined. Here, we found two mechanisms that regulate SGK1 protein abundance; both of them stem from the amphipathicity of the Nt α‐helix. One involves association of SGK1 to intracellular organelles: ER, LDs and autophagosomes, the other recruits various types of chaperones, illustrated in Figure 7. Previously, we showed that SGK1 binds to the ER where the ERAD ubiquitinates and degrades SGK1.^17^ Here we found that other organelles also recruit SGK1 and its survival varies according to the organelle type: LDs stabilize SGK1 doubling the half‐life in culture and in mouse tissues. In contrast, high flux of autophagosomes directs a fraction of SGK1 to lysosomal degradation reducing steady‐state level of SGK1. The mechanism underlying SGK1 captured by autophagosomes is likely mediated by ER‐phagy. Starvation and other stress conditions promote fragmentation of ER and capture by autophagosomes using receptors such as FAM134B and TEX264. SGK1 tethered to the cytosolic side of the ER co‐localizes with these receptors promoting recruitment into phagosomes. Conserved GIM‐like motifs in SGK1 carboxyteminus could further assist the process by binding GABARAPL1 and weakly GABARAPL2 in non‐starving cells.

Association to intracellular membranes is driven by the Nt α‐helix that hides hydrophobic residues into the lipid environment of membranes to protect SGK1 from degradation. The association does not require a protein receptor or special lipids; it depends entirely on the physical properties of the α‐helix demonstrated by SGK1 incorporation into artificial liposomes in vitro. However, large hydrophobic side chains are required for binding to LDs; if those residues are substituted with small hydrophobic side chains, SGK1 cannot attach to LDs, though association to other intracellular organelles remains intact. This result agrees with the notion that bulky hydrophobic side chains detect and bind membrane packing defects that are unique to the surface of LDs.^23^ The most salient functional consequence of SGK1 association to LDs is stabilization with a doubling of the half‐life likely owing to low ubiquitin/proteasome activity compared with that of the ER and cytosol. For most LD proteins, the ubiquitination machinery involved in their degradation is not intrinsically associated to LDs that is, it takes place in the ER or cytosol.^41^ The calculated *t_1_*_/_ *_2_* value is an underestimation of SGK1 survival in the LD compartment because only a fraction of the total SGK1 localizes to LD thus, in these organelles, SGK1 survival is expected to be longer than 42 min. These observations were confirmed in mice by demonstrating higher steady state abundance of SGK1 in adipose tissues than in lean tissues. Accordingly, SGK1 signalling would be expected to increase in metabolically active organs such as liver, muscle and fat where the content of LDs largely increases under physiological adaptations and metabolic diseases (obesity, hepatic steatosis, lipodistrophy and diabetes mellitus type 2).^42^, ^43^, ^44^ Few questions not solved in this study are whether newly synthesized SGK1 is targeted directly to LDs or SGK1 already tethered to the cytosolic leaflet of the ER migrates by diffusion to nascent LDs through membrane bridges. In addition, whether SGK1 attached to LD is just a bystander or plays a role in the physiology of LD. Our results, however, are in agreement with the previous reports of SGK1 participating in differentiation and function of adipocytes and development of obesity.^45^, ^46^

SGK1 also integrates into autophagosomes but the effect on stability is by redirecting a fraction of SGK1 to lysosomes. The answer to the question of how SGK1 ends up in autophagosomes is likely provided by ER‐phagy that carries SGK1 with ER fragments engulfed by autophagosomes recognized by ER‐phagy receptors. Furthermore, a conserved GIM‐like motif in SGK1 associates with GABARAPL1 and weakly with GABARAPL2 suggesting that SGK1 could be directly recognized as cargo by autophagosomes. Although we did not investigate whether SGK1 plays a role in the life cycle or biology of autophagosomes, no changes in autophagy levels were observed when SGK1 was expressed in 293T or in mice adipocytes. This differs from a previous report where a constitutively active SGK1‐S422D reduced autophagy in muscles of a transgenic mouse,^47^ likely because here, we used wildtype SGK1 rather than constitutively active. The results do not support cytosolic aggregates of SGK1 captured through ubiquitin‐dependent selective autophagy because ubiquitinated SGK1 did not form insoluble cytosolic aggregates that are the preferred cargo of selective autophagy.

The other system that determines SGK1stability is the protein quality control that we found interactive also with the Nt of SGK1. This motif recruits a wide range of chaperones, foldases and holdases that recognize the α‐helix as an unfolded polypeptide or as a mislocalized transmembrane domain. These chaperones were identified by crosslinking conducted with two different chemical reagents and spacer lengths: both recovered the same types of chaperones associated with full length SGK1 but not with ∆60SGK1 highlighting that the recruiting motif is the amphipathic α‐helix. Hsp90 and CDC37—that form a functional complex—were the exception because CDC37 selectively recruits client kinases binding to the catalytic domain.^35^, ^36^ We demonstrate that Hsp90/CDC37 protects the catalytic activity of SGK1 and confirm that inhibition of Hsp90 by geldanamycin reduces the phosphorylation levels of NDRG1. The functional significance of interactions with other heat shock proteins was demonstrated by the increased stability and abundance of SGK1 in the presence of functional Hsp70. A different type of quality control proteins that do not require ATP and express less abundantly than the Hsp family were identified: Bat3/Bag6 and SGTA. These two proteins together with UBL4A and TRC35 form a complex that recognizes exposed transmembrane domains in the cytosol. From the four complex components, only Bat3 and SGTA directly make contact with clients indeed, only those two proteins were identified, underscoring the specificity of the results. Interactions with SGTA stabilized SGK1 whereas Bat3 promoted ubiquitination and degradation. These findings were unexpected because those chaperones mainly assist proteins with terminal transmembrane domains in posttranslational insertion into the ER membrane, and also ubiquitinate and remove transmembrane proteins mislocalized in the cytosol.^37^, ^38^, ^39^, ^40^ SGK1 does not conform to either of the canonical features of Bat3/SGTA clients making evident that substrates different from those previously described can also be recruited. It is likely that Bat3 and SGTA misrecognize the amphipathic α‐helix of SGK1 as a potential transmembrane substrate delivering it to the ER where rather than facilitating insertion across the ER membrane SGK1 associates as a monotopic protein. Of note, none of the crosslinking experiments isolated TRC40 (40 kDa subunit of the transmembrane domain recognition complex, also known as ASNA1),^48^ which is the component of the Bat3 complex that directly transfers substrates to translocation receptors in the ER membrane.

How SGK1 may transition from chaperones to association with intracellular organelles and what are the consequences for SGK1 stability? The current findings are consistent with SGK1 recruiting chaperones early in its biogenesis, as the hydrophobic α‐helix is the first segment of the protein to emerge from ribosomes. The chaperones Hsp90, Bat3/SGTA and chaperonins are associated with ribosomes and engage with clients early during synthesis.^40^ Many proteins do not need chaperones after completion of synthesis as proper folding hides the hydrophobic patches. In the case of SGK1, exposure of the hydrophobic α‐helix persists after synthesis is completed; therefore, SGK1 maintains association with chaperones that either deliver the protein to the ubiquitin/proteasomal system for degradation or hand it over to other members of the quality control system. This agrees with the wide range of chaperone classes found in the crosslinking experiments. Ultimately, SGK1 is largely degraded in the cytosol. A fraction exchanges chaperones for the hydrophobic environment of lipid membranes. The α‐helix may tether directly to various organelles or may migrate by diffusion from the ER to LDs and autophagosomes. Though the association of SGK1 to membranes may be promiscuous, the amount present in a compartment depends on the degradative activity distinct to each organelle: ERAD in the ER, lysosomal proteolysis in autophagosomes and low proteolysis on the surface of LDs.^41^

The strict control of SGK1 abundance is frequently disrupted in pathological conditions such as in malignant cells where SGK1 expression is frequently increased, a property that extends to cells lines derived from malignant tumours as illustrated in the screening conducted here (Figure S3B).^49^, ^50^ High expression of SGK1 mediates resistance to PI3K inhibition in cancer cells where suppression of SGK1 or dual suppression of AKT/SGK1 might be useful for treatment. Our results revealed unsuspected ways to decrease SGK1 abundance by either targeting protein chaperones or disrupting SGK1 association to intracellular membrane, both of them are amenable to modulation for therapeutic purposes: drugs directed to chaperones^51^ and reduced calorie intake respectively. Whereas excess nutrients promote formation of LDs increasing SGK1 abundance, starvation decreases SGK1 by rerouting a fraction to degradation through autophagosomes and lysosomes. It has been recently shown that the efficacy of PI3K‐mTORC1 signaling axis suppression in tumors can be enhanced with diets that decrease insulin levels or mimic starvation.^52^, ^53^ Through the mechanisms described here, such treatment is expected to decrease SGK1 abundance, potentially contributing to the improved response to PI3K inhibitors in cancer cells and to lessen activation of proinflammatory pathways in immune cells.

## CONFLICT OF INTEREST

The authors confirm that there are no conflicts of interests.

## AUTHOR CONTRIBUTION

**Madiha Javeed Ghani:** Formal analysis (lead); Investigation (lead); Methodology (equal); Validation (lead); Visualization (lead); Writing – original draft (equal); Writing – review & editing (supporting). **Wenxue Gu:** Formal analysis (supporting); Investigation (equal); Methodology (supporting); Writing – original draft (supporting). **Zhuyuan Chen:** Formal analysis (supporting); Investigation (supporting); Methodology (supporting); Validation (supporting); Writing – original draft (supporting). **Cecilia M Canessa:** Conceptualization (lead); Formal analysis (supporting); Funding acquisition (lead); Project administration (lead); Supervision (lead); Writing – original draft (equal); Writing – review & editing (equal).

## Supporting information

Figure S1

Figure S2

Figure S3

Figure S4

Table S1

Table S2

Table S3

Supplementary Material

## Data Availability

The data that support the findings of this study are available from the corresponding author upon reasonable request.
