# Supplementary figures and images for: Lipid droplets and autophagosomes together with chaperones fine‐tune expression of SGK1
Source: J Cell Mol Med. 2022 Apr 8;26(10):2852–65. doi: 10.1111/jcmm.17300 (PMC9097849; doi:10.1111/jcmm.17300)

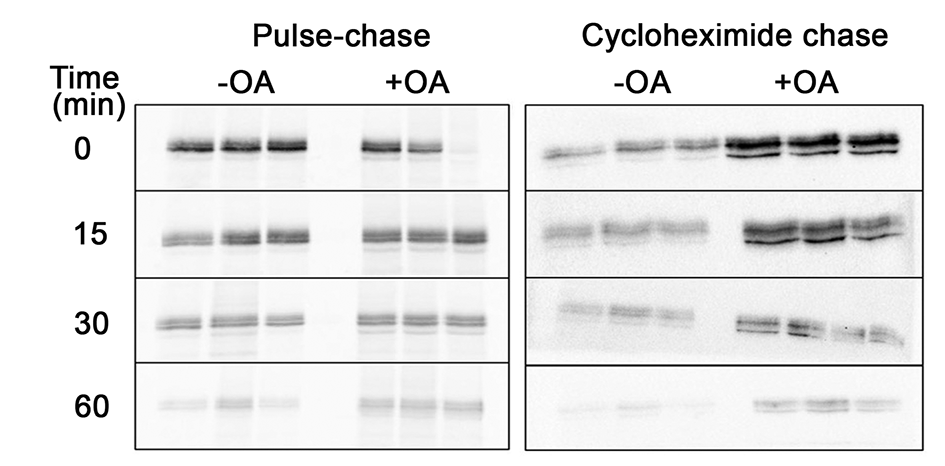

Supplement: Supplementary file 1 — Figure S1 [file JCMM-26-2852-s006.tif]

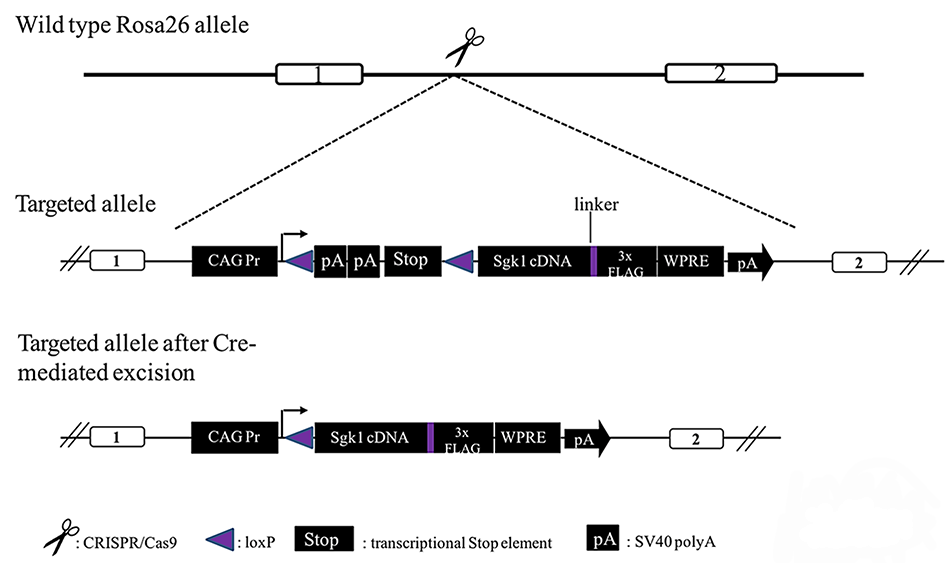

Supplement: Supplementary file 2 — Figure S2 [file JCMM-26-2852-s007.tif]

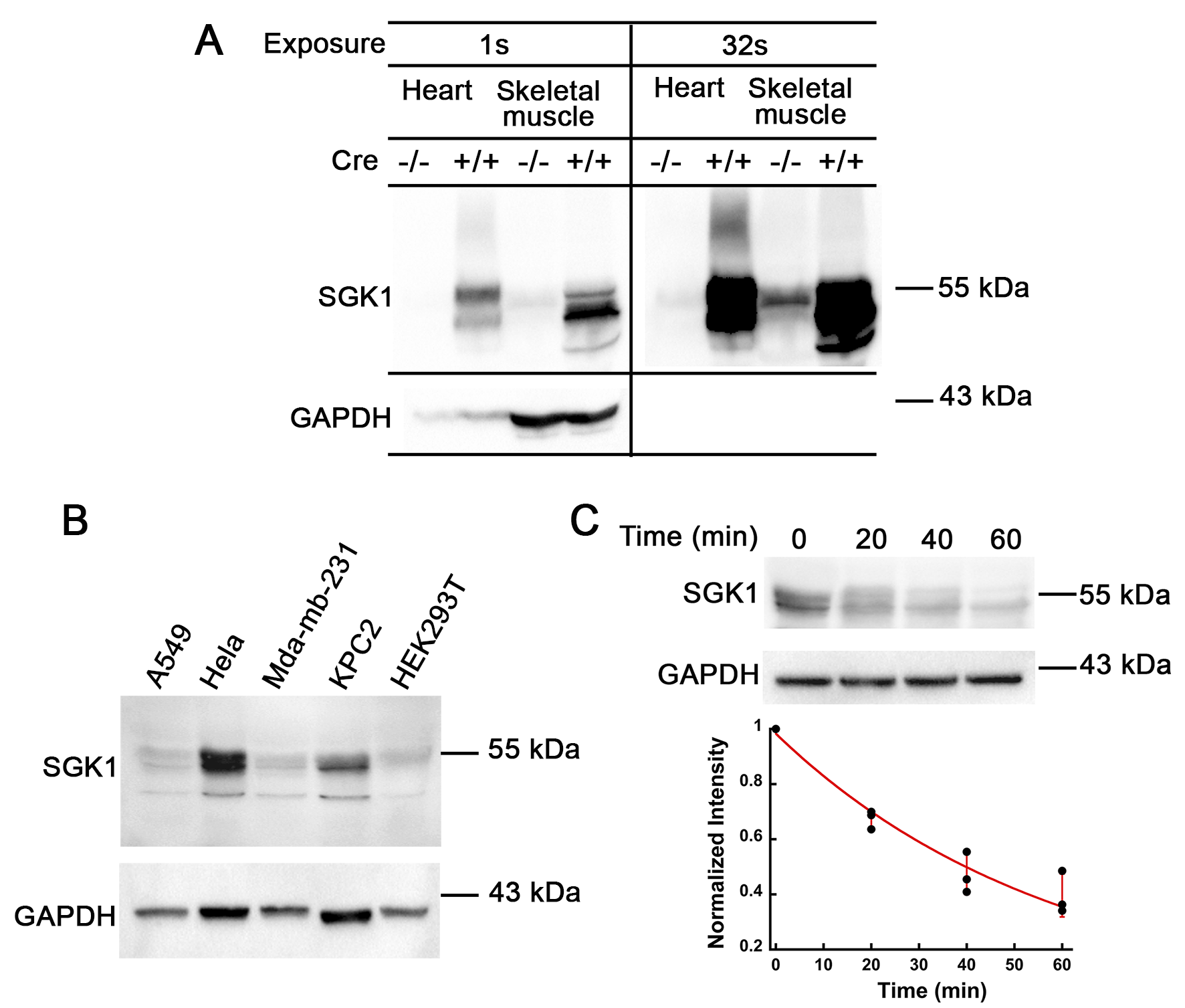

Supplement: Supplementary file 3 — Figure S3 [file JCMM-26-2852-s004.tif]

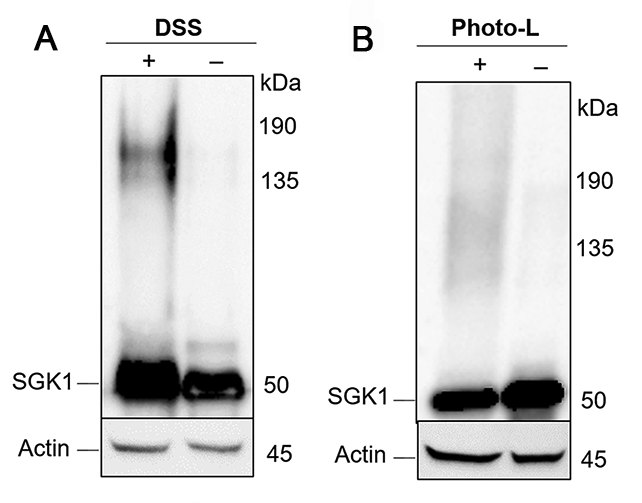

Supplement: Supplementary file 4 — Figure S4 [file JCMM-26-2852-s002.tif]
