# Supplementary material for: Lipid droplets and autophagosomes together with chaperones fine‐tune expression of SGK1
Source: J Cell Mol Med. 2022 Apr 8;26(10):2852–65. doi: 10.1111/jcmm.17300 (PMC9097849; doi:10.1111/jcmm.17300)
Supplement: Supplementary file 5 — Table S1 [file JCMM-26-2852-s008.docx]

| **Δ60SGK1** | | | |
| --- | --- | --- | --- |
| **DSS** | | **CONTROL** | |
| **Protein** | **Score** | **Protein** | **Score** |
| **Δ60SGK1** | 537.19 | **Δ60SGK1** | 92.12 |
| Hsp cognate 71 | 80.48 |  |  |
| Hsp 70 like | 63.08 | Hsp 70-like | 8.05 |
| Hsp 90 | 38.05 |  |  |
| CDC37 | 14.33 |  |  |
| T-complex protein 1 subunit α | 16.40 |  |  |
| T-complex protein 1 subunit β | 9.36 |  |  |
| DnaJ homolog subfamily A1 | 7.96 |  |  |
| T-complex protein 1 subunit γ | 7.78 |  |  |
| T-complex protein 1 subunit δ | 7.73 |  |  |
| T-complex protein 1 subunit ε | 6.29 |  |  |

**TABLE S1**
