# Supplementary material for: Lipid droplets and autophagosomes together with chaperones fine‐tune expression of SGK1
Source: J Cell Mol Med. 2022 Apr 8;26(10):2852–65. doi: 10.1111/jcmm.17300 (PMC9097849; doi:10.1111/jcmm.17300)
Supplement: Supplementary file 6 — Table S2 [file JCMM-26-2852-s001.docx]

| **SGK1** | | | |
| --- | --- | --- | --- |
| **PHOTO-LEUCINE** | | **CONTROL** | |
| **Protein** | **Score** | **Protein** | **Score** |
| Hsp 70-1A/1B | 471.24 | Hsp 70-1A/1B | 284.62 |
| Hsp cognate 7-1 | 373.43 | Hsp cognate 7-1 | 276.43 |
| Hsp 90-β | 345.85 | Hsp 90-β | 246.92 |
| Hsp 90-α | 306.85 | Hsp 90-α | 202.97 |
| **SGK1** | 287.16 | **SGK1** | 286.80 |
| Hsp 60 | 285.61 | Hsp 60 | 203.55 |
| Hsp 70 1-like | 162.61 |  |  |
| Hsp 70 related protein 2 | 154.95 | Hsp 70 related protein 2 | 133.80 |
| T-complex protein 1 subunit α | 108.59 | T-complex protein 1 subunit α | 127.21 |
| Hsp 75 | 77.31 |  |  |
| T-complex protein 1 subunit ζ | 70.01 | T-complex protein 1 subunit ζ | 44.22 |
| Hsp105 β | 68.39 | Hsp 105 β | 57.03 |
| T-complex protein 1 subunit δ | 68.22 | T-complex protein 1 subunit δ | 81.28 |
| T-complex protein 1 subunit ε | 58.85 | T-complex protein 1 subunit ε | 72.15 |
| Hsp 70-4 | 51.41 |  |  |
| T-complex protein 1 subunit η | 50.11 | T-complex protein 1 subunit η | 82.20 |
| FKBP4 | 38.87 |  |  |
|  |  | T-complex protein 1 subunit γ | 75.31 |
|  |  | T-complex protein 1 subunit ζ |  |
|  |  | T-complex protein 1 subunit β | 102.18 |

**TABLE S2**
