# Supplementary material for: Lipid droplets and autophagosomes together with chaperones fine‐tune expression of SGK1
Source: J Cell Mol Med. 2022 Apr 8;26(10):2852–65. doi: 10.1111/jcmm.17300 (PMC9097849; doi:10.1111/jcmm.17300)
Supplement: Supplementary file 7 — Table S3 [file JCMM-26-2852-s005.docx]

**Table S3. Quantification of Confocal Imaging Data.** Where R=1, perfect colocalization; R=0, no colocalization; R= -1, complete lack of colocalization; M1 & M2=1, complete overlap. In each scatterplot, C1: Channel red; C2: Channel green; M1: Fraction of C1 overlapping C2; M2: Fraction of C2 overlapping C1.

| No. | Sample | Pearson’s Correlation Coefficient (R) | Manders’ Coefficient M1 | Manders’ Coefficient M2 |
| --- | --- | --- | --- | --- |
| Figure 1D | SGK1:Calnexin | 0.964 | 0.976 | 0.918 |
|  | SGK1:GALNT2 | 0.434 | 0.082 | 0.842 |
|  | SGK1:Hsp60 | 0.121 | 0.0.029 | 0.011 |
| Figure 2A | DGAT2 (Control) | 0.885 | 0.822 | 0.798 |
|  | DGAT2 (OLA) | 0.884 | 0.947 | 0.917 |
| Figure 2B | SGK1:LD | 0.587 | 0.537 | 0.748 |
|  | SGK1:Calnexin | 0.723 | 0.856 | 0.852 |
| Figure 5E | SGK1:FAM134B (Control) | 0.995 | 0.996 | 0.999 |
|  | SGK1:GFP-LC3 (Control) | 0.917 | 0.995 | 0.991 |
|  | SGK1:FAM134B (Rapamycin+BAF) | 0.980 | 0.994 | 0.998 |
|  | SGK1:GFP-LC3 (Rapamycin+BAF) | 0.896 | 0.988 | 0.979 |
| Figure 5F | SGK1:TEX264 (Control) | 0.988 | 0.996 | 0.999 |
|  | SGK1:GFP-LC3 (Control) | 0.968 | 0.943 | 0.999 |
|  | SGK1:TEX264 (Rapamycin+BAF) | 0.99 | 0.989 | 0.998 |
|  | SGK1:GFP-LC3 (Rapamycin+BAF) | 0.923 | 0.944 | 0.996 |
| Figure 5H | SGK1:GABARAPL1 | 0.865 | 0.839 | 0.789 |
