# Supplementary material for: Lipid droplets and autophagosomes together with chaperones fine‐tune expression of SGK1
Source: J Cell Mol Med. 2022 Apr 8;26(10):2852–65. doi: 10.1111/jcmm.17300 (PMC9097849; doi:10.1111/jcmm.17300)
Supplement: Supplementary file 8 — Supplementary Material [file JCMM-26-2852-s003.docx]

**Material and Methods**

**Cell culture**. HEK293T cells (ATCC,CRL-11269), AD-293 (Stratagene# 240085), Hela (ATCC,CCL-2), A549 (ATTC, CCL-185), Mda-mb-232 (ATCC, CRM-HTB-26), and KPC2 (gift from Dr. Charles David, Tsinghua University) were cultured in Dulbecco’s Modified Eagle’s Medium (Gibco#11995040) with 10% Fetal Bovine Serum (Corning#35010CV) in a humidified incubator with 5% CO_2_ at 37^o^C.

**Plasmids, transfection and antibodies**. All constructs were inserted in pcDNA3.1 plasmid. QuickChange Kit (200521 Agilent, USA) was used for mutagenesis. Plasmid transfection was performed with Lipofectamine2000 (Invitrogen#11668019). Antibodies: monoclonal anti-HA (SantaCruz Biotechnologies#sc7392), anti-FL (Abmart# 26D11-3B9), anti-V5 (ThermoScientific#R960-25), rabbit monoclonal SGK1 (Cell Signaling#D27C11), Phospho-NDRG1, CDC37 and β-actin (Cell Signaling Technology#3217, 4793, 4967), HSP90 (EnzoBiochem#ADI-SPA-846-D), GAPDH mab-HRP conjugated (EasyBioTech.com #BE0034), FlagM2 mab-HRP conjugated (SIGMA#A8592), rabbit mab anti-Ubiquitin (R&D Systems MAB8595). Agarose-TUBE beads (UM401, LifeSensors, USA) for affinity purification of ubiquitinated proteins. Secondary antibodies: goat anti-mouse IgG, Horseradish peroxidase conjugate (Novex A16072, Life Technologies), goat anti-rabbit IgG, Horseradish peroxidase conjugate (Novex G- 21234 Life Technologies), goat anti-mouse IgG conjugated-AlexaFlour 488, AlexaFlour 594 or AlexaFlour 667 (ThermoScientific#A11017, A11020, A21245).

**Generation of SGK1-FL transgenic mice.** Fertilized eggs of wild-type C57BL/6 mice (Jackson Laboratory, USA) were co-injected with sgRNA targeting the Rosa 26 locus, CRISPR associated protein 9 (Cas9), and a construct containing full-length canonical mSGK1-3xFLAG under CAG promoter. LoxP sites flanked the transcriptional stop sequence and 1800 pb arms of identical sequences of the Rosa 26 site were added for homologous recombination (Figure S2). To activate ubiquitous expression of SGK1 in transgenic animals they were crossed with E2A-Cre (B6.FVB-Tg(EIIa-Cre) C5379Lmgd/J) mice (003724, The Jackson Laboratory, USA). Animals were bred to obtain and maintain homozygous SGK1-3xFlag. Mice were fed a normal diet and kept in standard conditions. The Institutional Animal Care and Use Committee of Tsinghua University approved all procedures.

**RNA isolation, cDNA synthesis and quantitative PCR**. Mice were anaesthetized with Avertin and perfused with PBS through an aortic canula. Dissected tissues were divided into two parts. One was for isolation of total RNA with TRIzol reagent and the other for extraction of proteins. Single strand cDNA synthesis was primed with Oligo (dT) using ProtoScript II first strand cDNA synthesis kit (NEB# E6560S). Quantificationss of HPRT1 and GAPDH mRNA were performed according to protocol describes by Livak et al.^18^ and using SYBR Selected Master Mix (Applied Biosystems#4472908). qPCR primers:

tgSGK1-F:5’-GCAGCCCAAGCTAGATCGAATT-3’;

tgSGK1-R:5’-TAGGTGTTGCTGGCAATCTTCT-3’;

mHPRT-F:5’-TCAGTCAACGGGGGACATAAA-3’;

mHPRT-R:5’-GGGGCTGTACTGCTTAACCAG-3’;

mGAPDH-F:5’-AACTTTGGCATTGTGGAAGG-3’;

mGAPDH-R:5’-GGATGCAGGGATGATGTTCT-3’

qPCR primers for amplification of endogenous human SGK1 in Hela cells:

hSGK1-F:5’-GCAGAAGAAGTGTTCTATGCAGT

hSGK1-R:5’-CCGCTCCGACATAATATGCTT

hGAPDH-F:5’-ACAACTTTGGTATCGTGGAAGG

hGAPDH-R:5’- GCCATCACGCCACAGTTTC

**Chemical- and Photo-crosslinking**. SGK1 or ∆60SGK1 transfected HEK293T were grown on ten 100 mm dishes. 2 mM disuccinimidyl suberate (ThermoScientific#21655) was applied for 30 min. Crosslinking solution was replaced by quenching buffer (mM): 25 Tris, pH 7.4 and 150 NaCl for 15 minutes followed by cell lysis. For photo-crosslinking, medium was replaced with Leu/Met-free DMEM (Gibco#30030) supplemented with 10 mM Photo-Leucine (ThermoScientific#22610) + 2 mM L-Methionine for 18-20 hours. Experimental samples were exposed to 365 nm UV light supplied by an 8W lamp at 1 cm distance for 15 minutes. Control samples were processed identically except Photo-Leucine was not added. After crosslinking, cells were homogenized in TENT-1% buffer (mM): 1% Triton-X100, 5 EDTA, 150 NaCl, 50 Tris, pH 7.4, protease inhibitors (Roche#04693116001).

**Mass spectrometry analysis**. Crosslinked proteins resolved on SDS-PAGE were stained with Imperial Protein Stain (ThermoScientific#24615). Bands were excised and subjected to in-gel Trypsin digestion. For LC-MS/MS analysis, peptides were separated by gradient elution with a Thermo-Dionex Ultimate 3000 HPLC system, which was directly interfaced with a Thermo Scientific Q Exactive mass spectrometer. The MS/MS spectra from each LC-MS/MS run were searched against the human UniProt database using Proteome Discoverer. This was accomplished by matching the measured masses with masses predicted from the databases. Measured peptide masses closely matched to the predicted masses indicated a high likelihood that the protein was correctly identified. A q-value smaller than 1% for the Peptide Spectrum Match (PSM) was considered to be correct.

**Immunoprecipitation and immunoblotting.** Cells were homogenized in ice cold TENT 1% for 15 min. Whole-cell lysates were clarified by centrifugation at 10,000 rpm for 10 min at 4^o^C and supernatants incubated with anti-HA (88837, Pierce, USA) or anti-FL (A2220, Thermo Fisher Scientific, USA) magnetic beads for 2h at 4^o^C. Beads were collected and washed three times with TENT 1%. Proteins were eluted with reducing SDS-PAGE sample buffer and heated at 90^o^C for 3 min prior to loading onto 12% linear or 8% to 20% gradient SDS-polyacrylamide gels. Proteins were transferred to Immobilon-P membranes (IPVH00010, Millipore, USA) using Trans-Blot Transfer Apparatus (Bio-Rad Laboratories, USA). After blocking with 5% dry milk for 30 min, membranes were probed with primary antibodies for at least 1h, washed three times with Tris-buffered saline with 1% Tween-20. Membranes were incubated with secondary antibodies conjugated with horseradish peroxidase. Signals were developed with Immobilon Western chemiluminescent HRP substrate (P90720, MilliporeSigma, USA) according to the manufacturer’s instructions. Images were captured with ChemiDoc XRS+ and analyzed with ImageLab Software (Bio-Rad Laboratories, USA).

**Immunofluorescence microscopy and visualization of lipid droplets.** Cells grown on glass coverslips treated with a 0.01% solution of poly-D-lysine (P4707, SIGMA, USA) were fixed with 4% paraformaldehyde (MFCD00133991, Thermo Fisher Scientific, USA) in PBS for 30 min followed by washes with PBS and permeabilization with TENT-1%. Primary antibody was incubated for 1h at room temperature. After three washes with TENT-1%, secondary antibodies conjugated with Alexa Flour were incubated at room temperature for 1 hour. Cells were washed three times with TENT-1% and laid on glass slides over VECTASHIELD mounting medium with DAPI (Vector Laboratories, USA). Lipid droplets were induced by supplementing the medium with a mixture of albumin and oleic acid (O3008, SIGMA, USA) in a ratio of 6:1 for 12hs. After cells were fixed, LDs were visualized with BODIPY 480/515 (D3238, Thermo Fisher Scientific, USA) diluted in DMSO at a final concentration of 1 µg/mL incubated for 1 h, followed by two washes with PBS. Images were taken with a Nikon A1R HD25/A1R HD25 LSM microscope (Minato, Japan) and analyzed using NIS-Elements software.

**Co-immunoprecipitation.** Transfected HEK293T were lysed with solubilization buffer (mM): 150 NaCl, 20 sucrose, 10 Hepes, 2 MgCl2, 1 ATP, 5% Digitonin, and protease inhibitor cocktail (cOmplete, Roche) for 30 minutes on ice. Tagged proteins were captured with anti-HA magnetic beads or anti-FL agarose beads for 2-8 h at 4^o^C. Beads were washed three times with solubilization buffer and proteins eluted with SDS-loading buffer, and heated at 90^o^C for 3 min. Samples were run on SDS-PAGE gels and transferred to membranes.

**Inhibition of endogenous Hsp70.** SGK1 transfected 293T cells were treated with increasing concentrations of Azure C (0.5, 6,12, 25 µM) for 3 hours at 37^o^C. Cells were harvested and lysed with TENT-1% buffer supplemented with phosphatase inhibitor cocktail PhosSTOP (Roche) for 30 minutes on ice. Cell lysates were clarified by centrifugation at 10K rpm. Supernatants were heated with SDS protein loading buffer for 3 min at 90^o^C. Proteins were resolved on 12% SDS-PAGE and blotted on membranes. Membranes were incubated with 1:1000 dilution of mouse anti-FL and anti-β actin antibodies. Intensity of bands was examined with ImageLab software (BioRad) to calculate the IC_50_ value of Azure C.

**Inhibition of endogenous Hsp90.** SGK1 transfected 293T cells were treated with 5 µM Geldanamycin (9843, Cell Signaling, USA) at 37^o^C for 6 hs. Cells were harvested and lysed with TENT-1% supplemented with phosphatase inhibitor cocktail PhosSTOP (Roche) for 30 min on ice. Cell lysates were clarified by centrifugation at 10K rpm. Supernatants were heated with reduced SDS protein loading buffer for 3 min at 90◦C. Proteins were resolved on 12% SDS-PAGE and blotted on membranes. Membranes were incubated with 1:1000 mouse anti-P-NDRG1, mouse anti-FL, and anti-β actin antibodies.

**TritonX-114 phase partitioning of SGK1.** TritonX-114 (X114, SIGMA, USA) phase partitioning was performed using the multiple washing method previously described.^19^ Proteins in the aqueous and detergent fractions were acetone precipitated, quantified and 15µg/lane was loaded onto SDS-PAGE followed by western blotting with HA and actin monoclonals.

**Incorporation of SGK1 into liposomes.** Recombinant wild-type SGK1-HA-H8 and ∆60SGK1-HA-H8 were isolated from transfected HEK293T (10 and 3, 100 mm plates, respectively) using Ni-NTA agarose (QIAGEN) according to manufacturer instructions. Centrifugal Filter units (Amicon Ultra-4) were used for buffer exchange and concentration of eluted proteins. Liposomes were made according to a protocol previously published.^20^ Phospholipids (Avanti Polar Lipids, USA) in the following molar ratios and composition: 1-palmitoyl-2-oleoyl-sn-glycero-3-phosphocholine (POPC): 1-palmitoyl-2-oleoyl-sn-glycero-3-phosphoethanolamine (POPE): 1-palmitoyl-2-oleoyl-sn-glycero-3-phospho-L-serine (POPS)=7:2:1. A uniform population of liposomes was made by 21 passes through a 0.1 µm PC-membrane using Avanti-Mini-Extruder that yielded a normal unimodal distribution of 100 nm vesicles. Recombinant proteins were incubated with the liposomes in 1:1 molar ratio at 4^o^C for 3h. The mixture was dialyzed against 3 x1 L changes of buffer (150 mM NaCl, 5 mM EDTA, 20 mM Hepes, pH: 7.4). Step sucrose gradients were used to separate SGK1 and ∆60SGK1 incorporated in proteoliposomes from unincorporated proteins. 300 µl of the proteoliposome preparation was mixed with 200 µl of 2.2 M sucrose dissolved in dialysis buffer and placed in 2 mL polycarbonate centrifuge tubes. This mixture was overlaid with a 400 µl cushion of 0.75 M sucrose, and on top 100 µl layer of buffer without sucrose. Gradients were centrifuged for 2 hs at 240K g in a Beckman TLS-55 rotor. Three fractions were collected from the bottom and analyzed by western blotting with anti-HA antibody.

**Pulse-chase and cycloheximide chase**. 293T transfected with SGK1 wild type were supplemented with oleic acid-albumin solution for 12h while control samples were kept with serum-free DMEM. Cells were washed with Methionine and Cysteine free DMEM (21013, Gibco, USA) twice for 5 min to deplete methionine and cysteine. 100 µCi/mL of S35 (NEG009L005MC, Perkin Elmer-Cetus Life Sciences, USA) mix of L-Methionine and L-Cysteine (M9625, 168149, SIGMA, USA) was added for 30 min. Labeling solution replaced by chase medium (DMEM supplemented with 100 µg/mL of Cycloheximide (239764, SIGMA, USA), 0.63mg/mL L-Cysteine and 0.3mg/mL of L-Methionine. Cells were returned to 37^o^C in the incubator for 0, 15, 30, 60 min. Cells were washed with PBS, lysed with TENT-1% on ice, and prepared for immunoprecipitation with anti-HA magnetic beads. Gels were dried and exposed to a phosphorimager screen. Analysis was conducted with Typhoon trio+ multifunction scanning imaging system (GE Healthcare, USA). For cycloheximide only chase experiments, 200 µg/ml of cycloheximide was added to the medium at time zero and followed for the times indicated in the respected experiments. Chase was stopped by adding Triton-1% buffer. Equal amounts of protein from each time point were loaded on gels followed by western blotting to detect SGK1 and a loading control: GAPDH or actin. Band intensity was used to calculate t_1/2_ by fitting to a single exponential.

**Quantification of ubiquitinated SGK1.** 293T were transfected with pcDNA3.1(-) empty vector, SGK1-FL, and SGK1-FL:pCMV6 Entry-Bat3-Myc-DDK plasmids. After 20 hs, cells were treated with 30 µM PR-619 (DI40483, LifeSensors, USA) for 2 hours. Cells were lysed (with 1% Triton-X 100, 4 mM Phenanthroline, 30 µM PR-619, protease inhibitor cocktail) on ice for 30 min. Lysates were clarified by centrifugation at 10K rpm at 4^o^C for 5 min and with uncoupled agarose beads for 30 minutes at 4^o^C on a rocker platform. After removal of beads, a fraction of lysate was taken and quantified by BSA protein assay kit (23227, Thermo Fisher Scientific, USA) to serve as the input signal. Agarose-TUBE beads (UM401, LifeSensors, USA) were added to the remaining sample and incubated for 3hs. Beads were collected by centrifugation at 10K rpm for 5 min and washed three times with TBS-T. The final supernatant after ubiquitin affinity purification was used as the unbound fraction. TUBE beads were eluted with SDS sample buffer and boiled at 90^o^C. Samples were processed for SDS-PAGE and western blotting. Monoclonal anti-FL M2 Peroxidase conjugated and rabbit monoclonal anti-Ubiquitin antibodies were used to detect protein signals by western blotting.

**Quantification of colocalization in fluorescent microscopy images.** Colocalization analysis was performed to quantify colocalization of proteins of interest to subcellular structures. Original RGB confocal images captured at 100X magnification were processed for immunofluorescence colocalization using ImageJ software containing plug-ins "Colocalization Finder” and “JACoP”. The level of overlap between the two channels and the correlation within the overlap were simultaneously assessed by calculating Pearson’s correlation coefficient (R) and Manders’ coefficients (M1 and M2).^21^ Data denoising and background subtraction of the original confocal images were followed by automated thresholding to isolate signal in both channels. Scatterplots of red and green pixel intensities provided a qualitative measure of the degree of colocalization and intensity distribution of two channels. The value of R ranges from 1 to -1 (R=1, perfect colocalization; R=0, no colocalization; R= -1, complete lack of colocalization). While M1 and M2=1 means complete overlap. Summary of quantification data from all images is presented in Table S4.

**Statistical analysis**. Results are expressed as mean±SD from at least three independent experiments. Two-group comparison was performed with two-side Student’s t-test using. Multiple group comparisons were performed with one-way ANOVA. p-values < 0.05 were considered statistically significant using Graphpad software (San Diego, USA).
